# Supplementary figures and images for: Genomic diversity of bacteriophages infecting Rhodobacter capsulatus and their relatedness to its gene transfer agent RcGTA
Source: PLoS One. 2021 Nov 18;16(11):e0255262. doi: 10.1371/journal.pone.0255262 (PMC8601537; doi:10.1371/journal.pone.0255262)

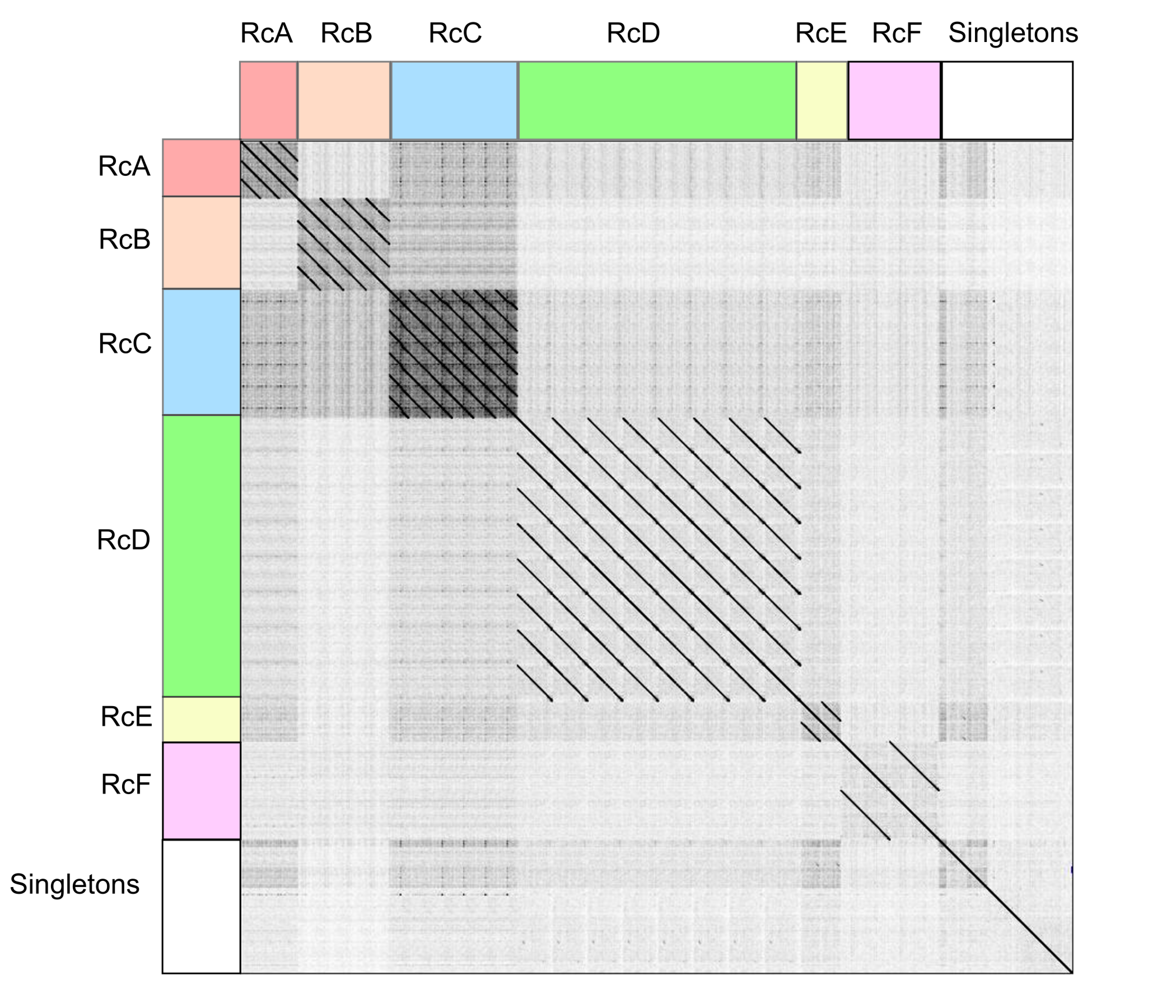

Supplement: S1 Fig — A dotplot comparison of the catenated genomes against themselves was created using Gepard [1]. Areas of clustering are color-coded to match the cluster colors on Fig 2. (TIF) [file pone.0255262.s001.tif]

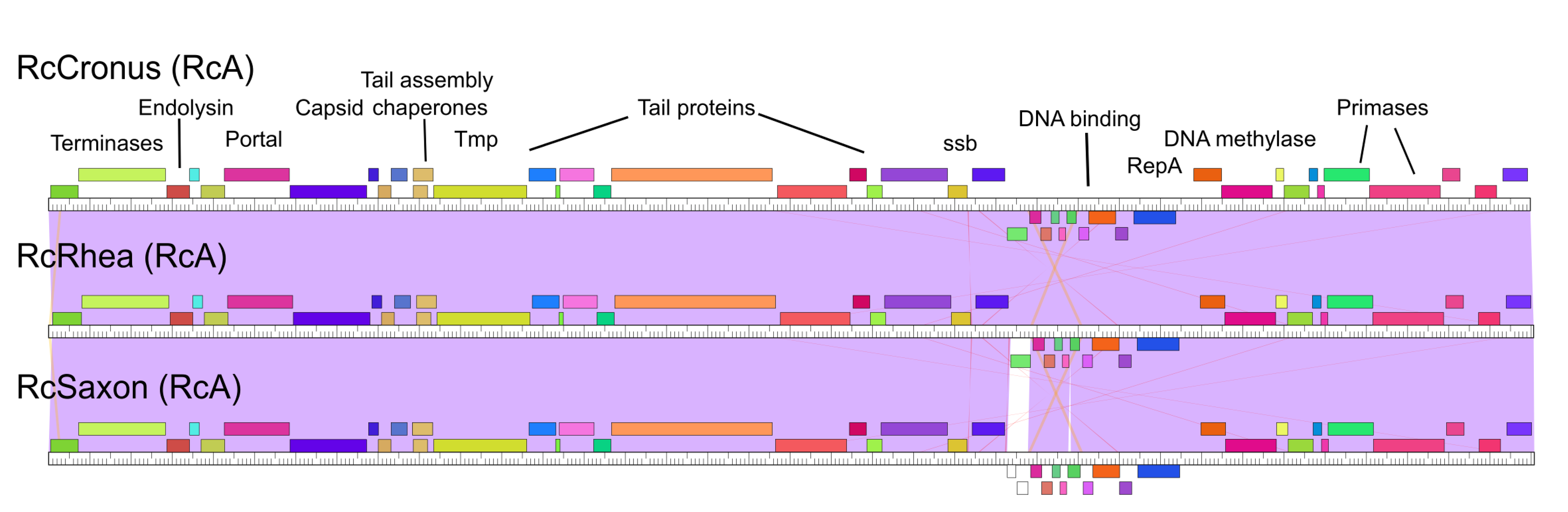

Supplement: S2 Fig — Genome maps of the RcA phages are shown. Pairwise nucleotide sequence similarities are displayed with spectrum-coloring between genomes, with violet representing greatest similarity and red the least similar, above a threshold E value of 10−3. Genes are represented as boxes above or below the genomes reflecting rightwards- and leftwards-transcription respectively. Genes are colored according to their phamily designations using Phamerator [31] and database Rhodobacter_capsulatus. (TIF) [file pone.0255262.s002.tif]

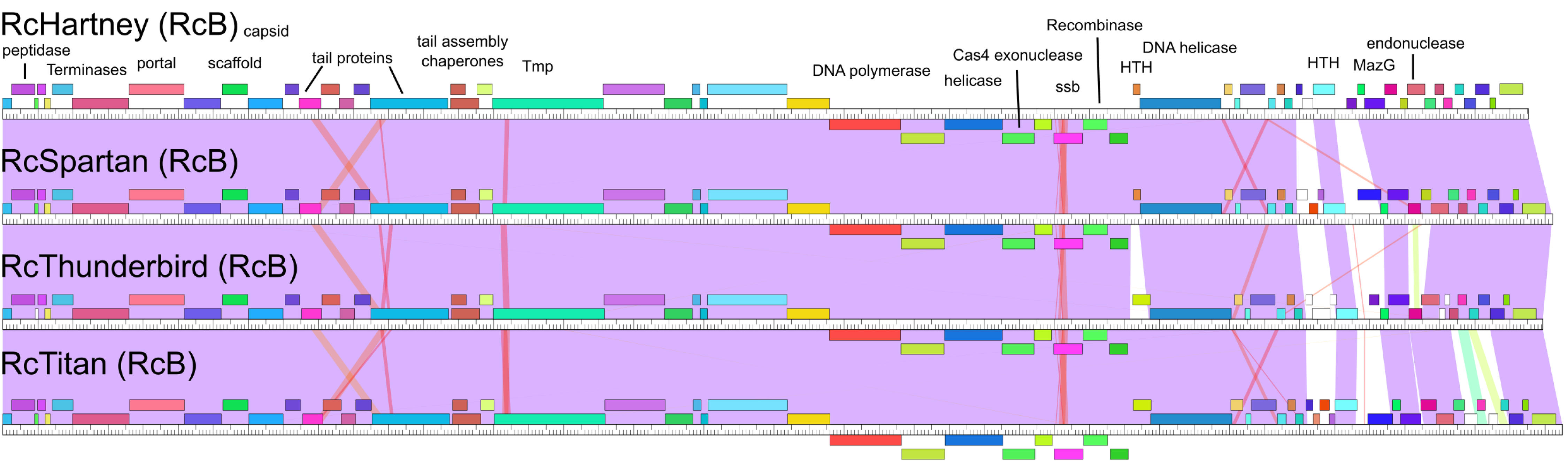

Supplement: S3 Fig — See S2 Fig for details. (TIF) [file pone.0255262.s003.tif]

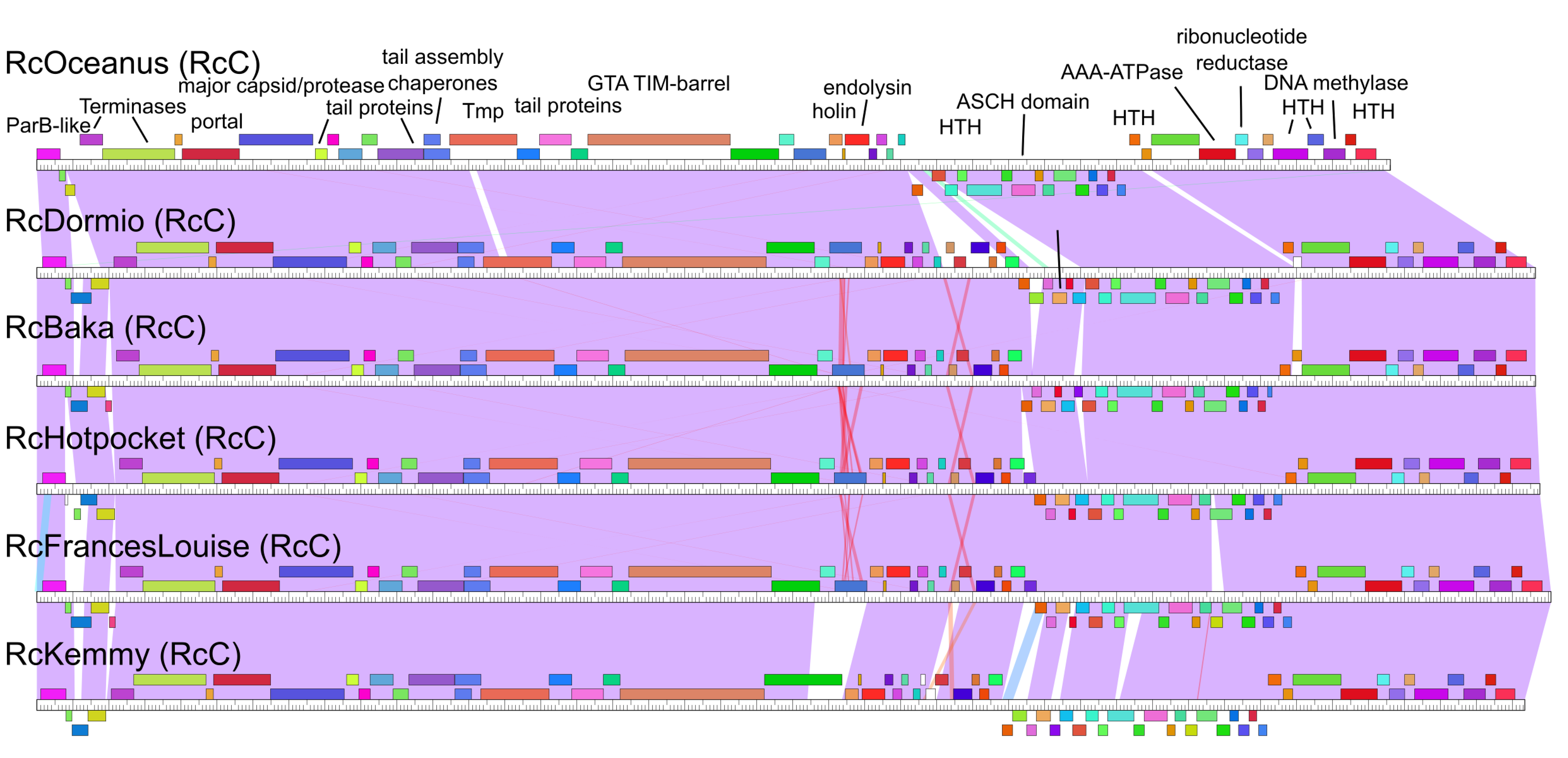

Supplement: S4 Fig — See S2 Fig for details. Areas where red lines appear between genome maps indicate the presence of repeat sequences. (TIF) [file pone.0255262.s004.tif]

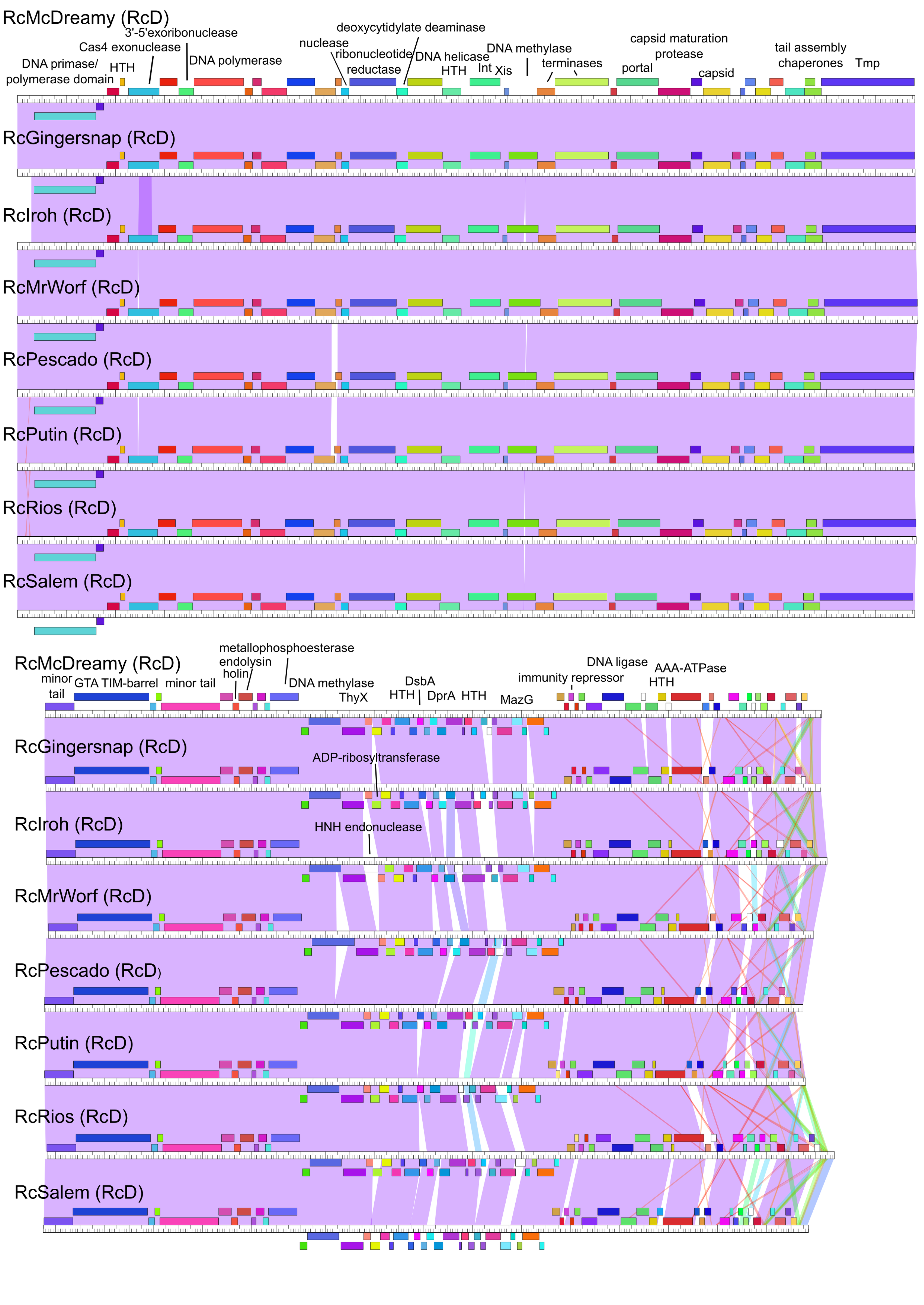

Supplement: S5 Fig — See S2 Fig for details. (TIF) [file pone.0255262.s005.tif]

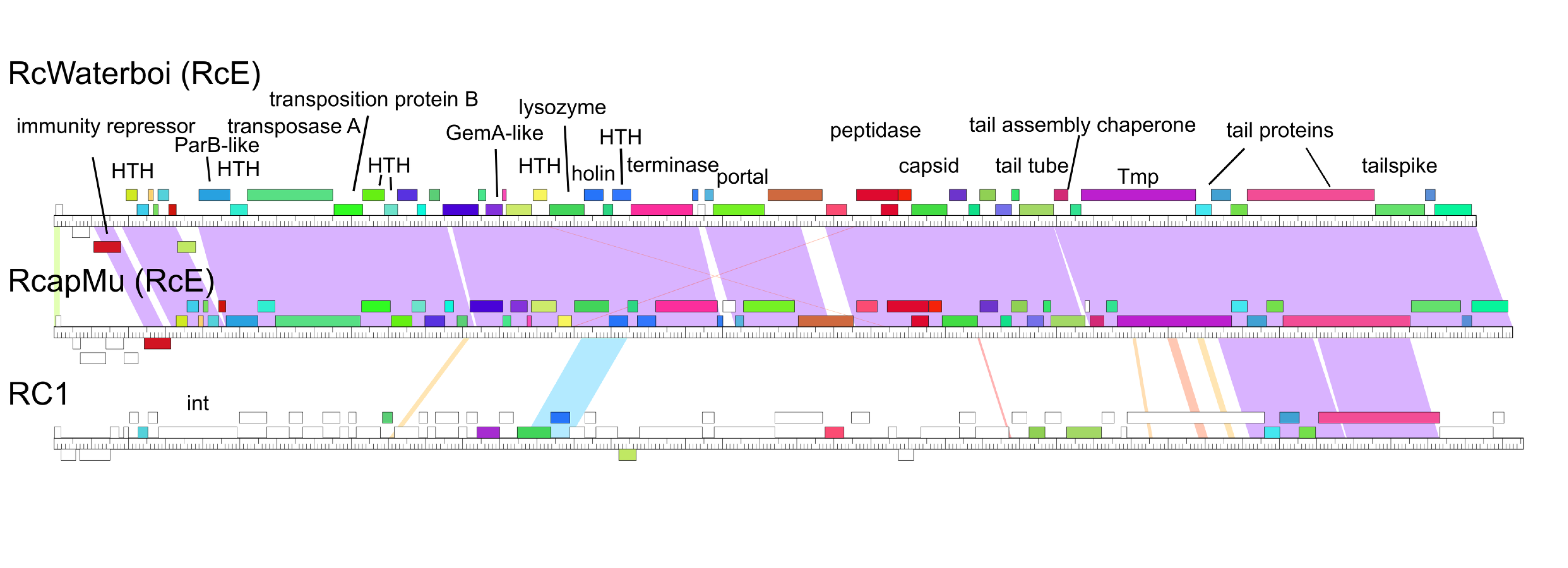

Supplement: S6 Fig — Genome maps of the RcE phages along with the singleton RC1 are shown. See S2 Fig for details. (TIF) [file pone.0255262.s006.tif]

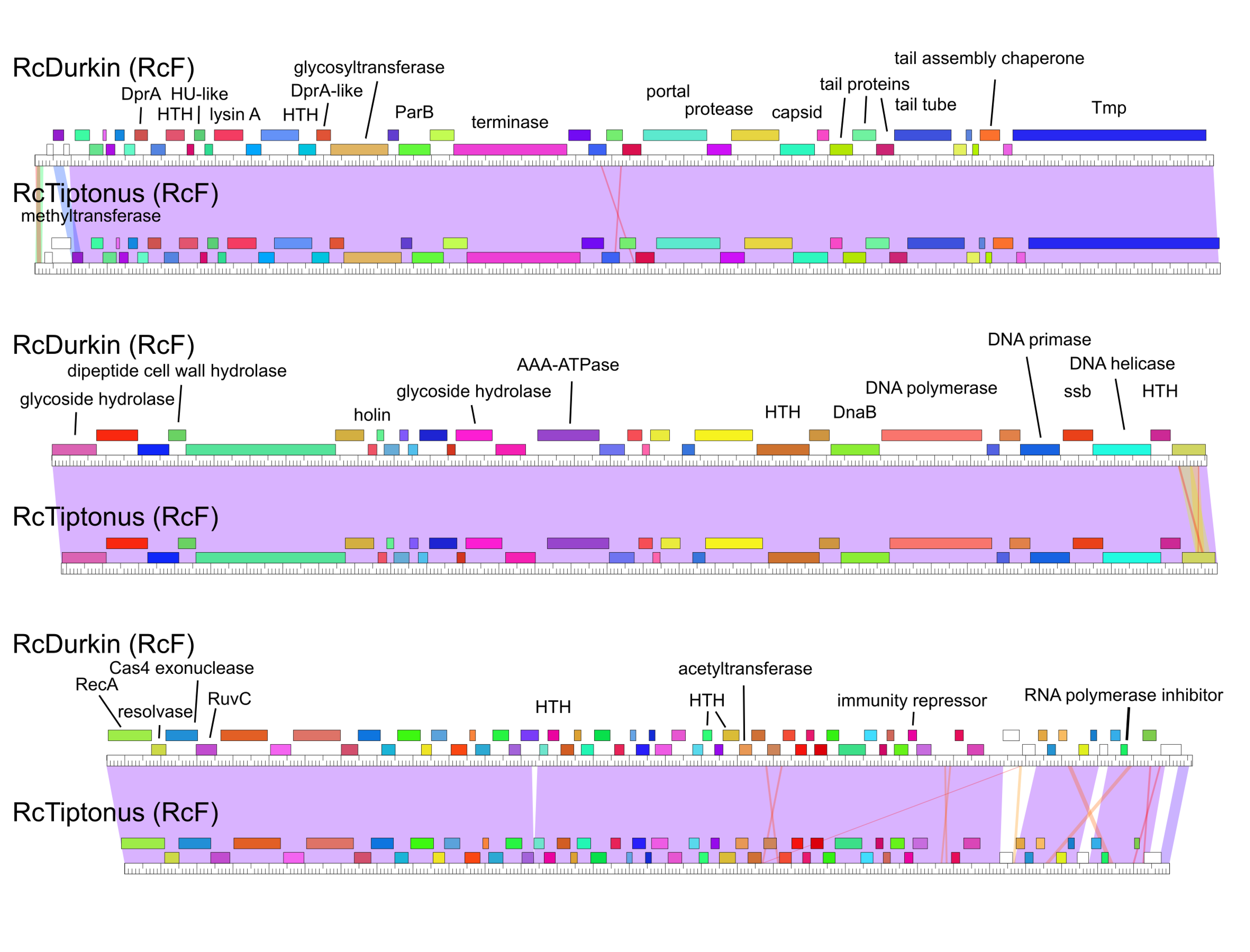

Supplement: S7 Fig — See S2 Fig for details. (TIF) [file pone.0255262.s007.tif]

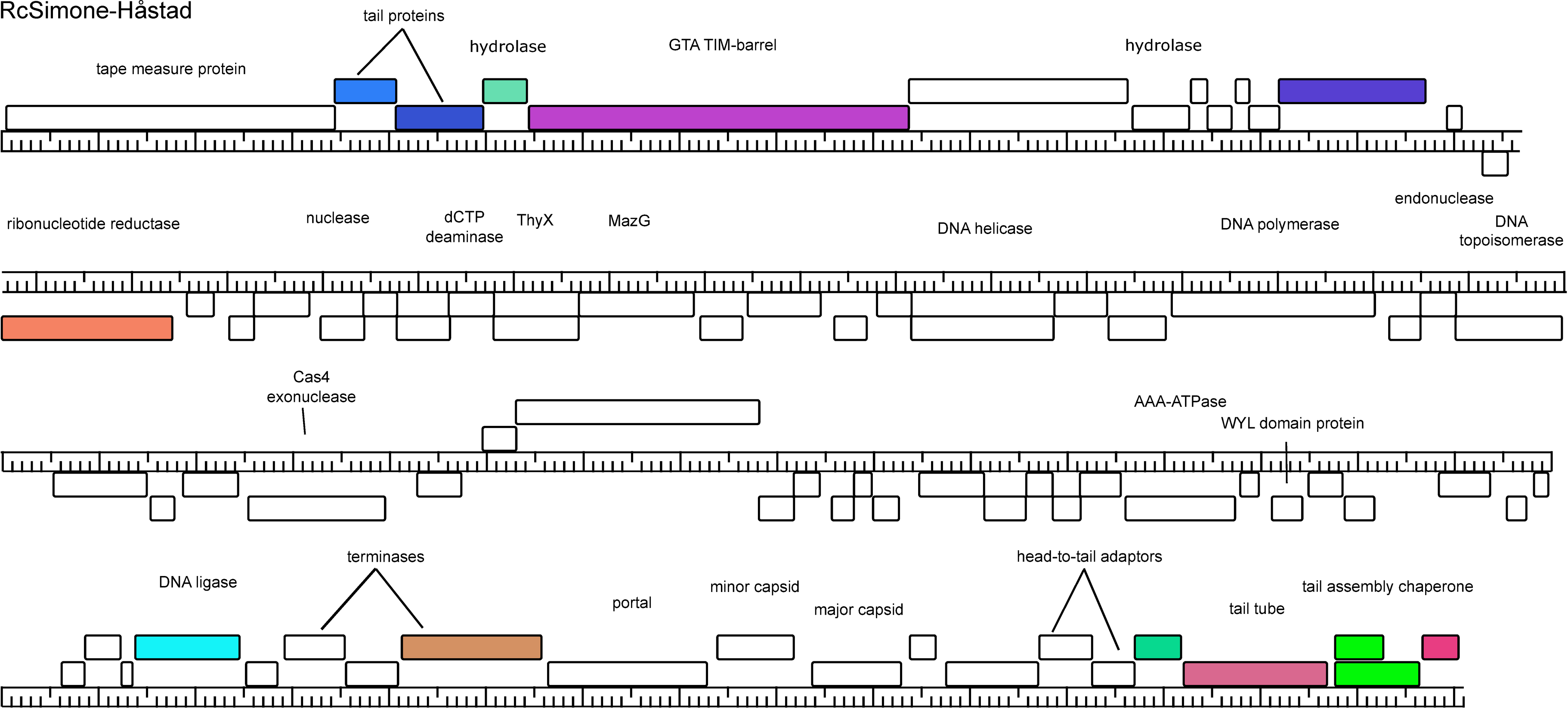

Supplement: S8 Fig — See S2 Fig for details. (TIF) [file pone.0255262.s008.tif]

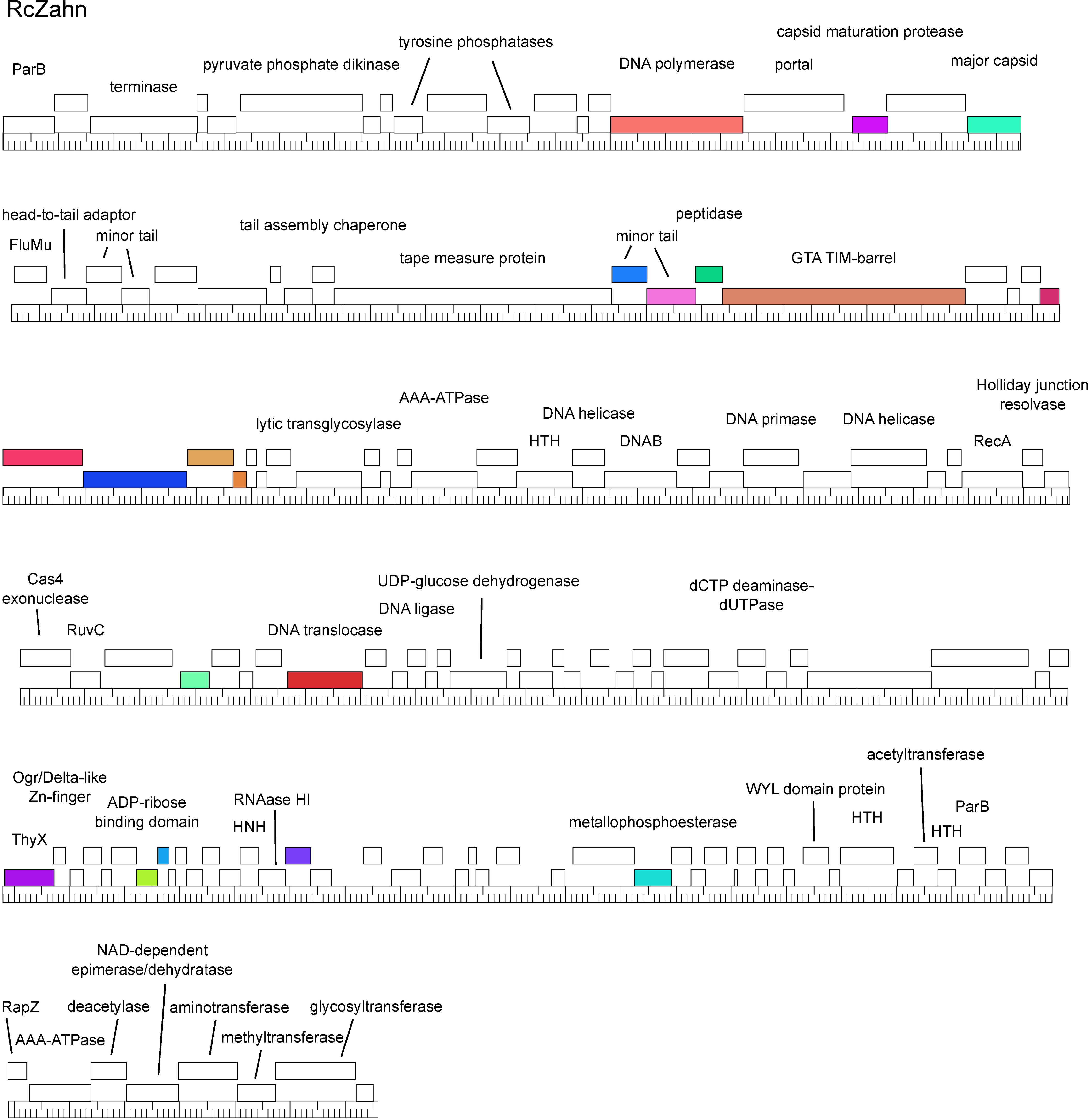

Supplement: S9 Fig — See S2 Fig for details. (TIF) [file pone.0255262.s009.tif]
