# Supplementary material for: Genomic diversity of bacteriophages infecting Rhodobacter capsulatus and their relatedness to its gene transfer agent RcGTA
Source: PLoS One. 2021 Nov 18;16(11):e0255262. doi: 10.1371/journal.pone.0255262 (PMC8601537; doi:10.1371/journal.pone.0255262)
Supplement: S1 File — (DOCX) [file pone.0255262.s010.docx]

Supporting Information Reference List

1. Krumsiek J, Arnold R, Rattei T. Gepard: a rapid and sensitive tool for creating dotplots on genome scale. Bioinforma Oxf Engl. 2007 Apr 15;23(8):1026–8.
