# Supplementary material for: Genomic diversity of bacteriophages infecting Rhodobacter capsulatus and their relatedness to its gene transfer agent RcGTA
Source: PLoS One. 2021 Nov 18;16(11):e0255262. doi: 10.1371/journal.pone.0255262 (PMC8601537; doi:10.1371/journal.pone.0255262)
Supplement: S2 File — (DOCX) [file pone.0255262.s011.docx]

**Supporting Information**

Class Rosters:

**SEA Lab 2015** (Phages: RcFrancesLouise, RcDormio, RcMcDreamy):

Seth Borrowman

Morgan Braun

Brandon Chang

Megan Dolan

Addison Ely

Emily Erdmann

Madeline Gibson

Samridh Gupta

Brooke Koebele

Julia Lennon

Alexandria Paradis

Robert Shafer

Ellen Stumph

Niyant Vora

Daniel Walski

Ling Zheng

**SEA Lab 2016** (Phages: RcPescado, RcMrWorf, RcGingersnap, RcIroh, RcPutin):

Marissa Alcala

Kristian Avise-Rouse.

Alecia Beagles

Janna Fitzgerald

Tulasi Jaladi

Jessica Keen

Zoephia Laughlin

Caroline Marchi

Ice Nie

Colin Page

Bailey Reichert

Morgan Reish

Andria Talavera

Sydnie Wells

Samantha Ziomek

**SEA Lab 2017** (Phages: RcHotPocket, RcBaka, RcWaterboi):

Zaain Ahmad

Meghan Bowler

Julia Chen

Jaeden Danko

Brooke Dominski

Megan Frederick

Lilia Garcia

Carlie Haagen

Greg James

Rosemary Josenkoski

Sydney Longfellow

Sommer Martin

Ria Patel

Andrew Runkle

Jory Vance

Julie Xu

**SEA Lab 2018** (RcHartney, RcKemmy, RcZahn, RcDurkin):

Emmett Agting

Vanessa Chapa

Matt Crosse

Ivy Do

Maeghan Eaker

Chris Eisenreich

Kirstin Kiper

Anjali Nimavat

Manish Pathuri

Jackson Rapala

Taima Saei

Kylie Spiegel

Saralexis Torres

Katie Vogler

Lane Warren

Saylor Williams

**Molecular Biology 2017:**

Christopher Johnson

Nitsueh Kebere

Tiffany Le

Winston Lee

Violet Pavlik

Jared Renfroe

Jessica Smith

Yash Thacker
